# Five challenges for disability-related research in sub-Saharan Africa

**DOI:** 10.4102/ajod.v3i2.149

**Published:** 2014-09-19

**Authors:** Leslie Swartz

**Affiliations:** 1 Department of Psychology, Stellenbosch University, South Africa

## Abstract

Disability research in contemporary sub-Saharan Africa is developing rapidly, and this is something to be celebrated. This article reviews some contemporary developments and suggests that there are five central, and interrelated, challenges for the field. These challenges – experience, expertise, enumeration, evidence, and expectations – go to the heart of thinking about disability research in sub-Saharan Africa. An optimistic but appropriately critical approach to addressing these issues is suggested.

## Introduction

There is good reason to feel both proud and optimistic about disability-related research in sub-Saharan Africa. I shall name a few examples of successes. The establishment and continued vitality of AfriNead (the African Network of Evidence to Action on Disability) is no small achievement. AfriNead has hosted a number of conferences and meetings attended by a range of researchers, scholars, and disability activists from Africa and further afield, with a special issue of the prestigious journal *Disability and Rehabilitation* devoted to AfriNead work (Mji *et al*. 2009, 2011). The *African Journal of Disability* (an AfriNead project) is up and running, and has gained official status as a recognised journal by the South African Department of Higher Education and Training within three years of it being tentatively established. The Southern Africa Federation on Disability (SAFOD) established and ran a research programme which had its challenges (not least of which was the untimely death of the late Alexander Phiri, the charismatic and hugely influential SAFOD leader), but which produced research – some of which has been reported in the latter journal – and built research capacity amongst disability activists (Swartz 2009; 2013). The Disability Studies Programme at the University of Cape Town has recently celebrated 20 years of postgraduate training in the field. Kwame Nkrumah University of Science and Technology (KNUST) in Ghana has a centre, CEDRES, devoted to disability research, which is a co-owner of the *African Journal of Disability*. The Centre for Disability and Rehabilitation Studies at Stellenbosch University, the other owner of the journal, has been involved in a number of research initiatives in a range of African countries. There are many other centres devoted to disability issues in a number of African contexts. In this special issue of the journal there are, furthermore, examples of successful disability research projects, some of them large-scale and multi-country.

There are important collaborations internationally. Notable amongst these collaborations is the long-standing work SINTEF (Stiftelsen for industriell og teknisk forskning) in Norway has carried out with the disability movement in various sub-Saharan countries. The reports on living conditions of disabled people in a number of African countries, all conducted together with SINTEF, are foundational to much of the research in the region. These reports can be downloaded from http://www.sintef.no/home/Technology-and-Society/Projects/Projects-SINTEF-TS-2006/Studies-on-living-conditions/. Similarly, Trinity College Dublin has worked on a number of collaborative projects in the region: see, for example https://global-health.tcd.ie/research/projects/APODD.php and http://www.equitableproject.org/. The Leonard Cheshire Disability and Inclusive Development Centre (http://www.ucl.ac.uk/lc-ccr), located at University College London, is playing an increasing role in collaborations in the region. There are many other examples. If disability research in southern Africa is to have a global impact, these collaborations are crucial.

The success and international visibility of the work many people are doing to develop disability research in our region was brought home to me recently when I was invited to give a talk at a prominent university in the USA, well known for its work on disability issues. Before I gave the talk, I met a well-known disability scholar and activist, and one of the first things he said to me was, ‘How is it that your university and others around you are getting disability issues so right?’ I felt pleased that this person knew about what we are doing in Africa, and more pleased that he was complimentary about our work: what emerged in our conversation was even more interesting. He was of the view that because of the constraints of an increasingly narrow, output-driven research environment in the United States of America (USA), it is becoming less and less possible for academics there to engage in deeply critical work which questions current hierarchies of knowledge and power. For this, it seems, he was looking to us – to African researchers working in contexts which I always think of as far more challenging than those in the USA. Not least amongst these challenges is the obvious one of a lack of resources, research infrastructure, and an enabling research environment, to which we must add the deep poverty and social exclusion of most people on whose behalf we commonly claim to be doing research. I was reminded of the wisdom of Jean and John Comaroff (2011) who in their book, *Theory from the south or how Euro-America is evolving towards Africa*, argue that scholars in Africa have long been dealing with challenges which are relatively new to some of their counterparts in wealthier countries – poverty and gross inequality, migration and refugee issues, environmental challenges and disasters, corruption and violence – partly because of the cultural encapsulation of many scholars in wealthier countries. The Comaroffs argue that because of this, and for other reasons, the days when scholars and researchers in the global South would be consumers and adapters of theory and expertise from the North are over: indeed, if the North wants to address its own emergent issues, the people to learn from may well be researchers embedded in global South contexts.

All of this is exciting, but also challenging. We may not yet be at a stage where we can claim that disability research in sub-Saharan Africa has come of age, but it is probably fair to say that we are in a process of growing up and are starting to be seen as growing up. And with this increasing maturity and substantially increasing visibility comes a growing responsibility. It may currently still be the case that, because of the dearth of information about disability issues on our continent, funders, international agencies, journal editors and reviewers, may work harder than they otherwise would to support research from our region. This is absolutely as it should be: it is simply outrageous that most of what we know about disability issues across the board comes from wealthier countries, when by far the majority of disabled people in the world live in low and middle-income countries (World Health Organization [WHO] & World Bank, 2011). Even small steps to redress the knowledge gap should be supported, and capacity must be developed. As more and more becomes known, even in small areas in the disability field, it is right also to expect more from researchers. The central question here is how do disability researchers in our region retain the distinctive strengths which come from working in our contexts, whilst at the same time developing the quality and depth of our work, and its ability to contribute to changing people’s lives for the better? There have been successes in this regard, notably the recent book by Brian Watermeyer (2012), arguably the most sophisticated book available internationally on the psychology of disablism, and a book embedded in Watermeyer’s experience in the disability field in South Africa. But how do we do more?

This is a large and a complex question, and not one than can be answered by a single author and in the confines of a single journal article. In order to begin to address the question in the remainder of this article I shall, therefore, briefly consider five interrelated challenges which I have encountered in my own attempts to develop disability research and disability research capacity in our region. For ease of presentation, I call five linked key challenges, the five Es: experience, expertise, enumeration, evidence, and expectations. I shall discuss each briefly.

## Experience

An important contribution of both the social model of disability (Swain *et al*. 2013) and of feminist disability studies (Garland-Thomson 2005) to how disability studies are thought of as a research discipline, is the placing of insider experience at the centre of how we understand and think about disability issues. Within previous models of disability – most prominently, that which is usually termed the medical model – non-disabled professionals were seen to have the expertise in the field of disability. Professional expertise and research was thought to be what was needed in order to understand disability best. This is no longer the case. First person accounts of experiences of disability and social exclusion are now common and thought important for any full understanding of disability (Couser 2009; 2012). There are good examples of the use of insider accounts in scholarship in our region (for example Human Rights Media Centre 2011; Moolman 2010), and scholarship discussing such accounts, amongst others, as discursive forms (Lipenga 2014). These experiential insider accounts are important in expanding what we know about disability, and also in changing the rules how we come to know about disability. For too long, such accounts were dismissed, and continue to be dismissed, as ‘mere anecdote[*s*]’, but there are important lessons to be learned from those actual stories told by disabled people (Swartz *et al*. 2012). For the purposes of this article, there are, however, three major potential problems with relying too heavily on such accounts.

Firstly, there is a problem with the assumption that insider knowledge is always representative of the views of all people of a certain group. To assume that the stories of six blind people in South Africa will, for example, tell the whole story of all blind people in South Africa is absurd. Clearly, we need methods which provide the valuable depth of these insider experiential accounts, but we also need methods which can provide some breadth on the basis of which we can generalise.

A second problem with over-valuing insider accounts is that such accounts may be incorrectly assumed simply to reflect the ‘truth’ about those whose accounts are portrayed. The reality is that every story anybody tells is profoundly affected by conventions, forms and tropes of stories: in life-writing about disability many stories are, for example, written according to the formula of portraying the disabled person as initially despairing and excluded, and then triumphing over adversity (Couser 2009; Swartz 2010). As the South African disability scholar Richards (2008) has noted, even in first-person or autoethnographic accounts, the ‘I’ doing the writing is not the same ‘I’ who had the experiences, even if they are ostensibly the same person. Every person reflecting on experience will filter this experience through a series of lenses, some of them conscious and some less conscious. In summary, then, research using experience is useful and important but not sufficient to tell us all we need to know about disability.

A third, and especially tricky, problem relates to how we value insider accounts concerns on what we make of individual people’s views on quality of inclusion and services they have received, and the need for services in the future. It is completely appropriate, and essential, to take personal stories into account when considering improving inclusion and participation and developing services. Such detailed accounts can provide much needed texture in how we think about inclusion and services (Mgwili & Watermeyer 2006). But there may be a bias in what gets published to favouring stories which are particularly interesting: few people wish to read stories which are boring and every day. Hence, stories which make recommendations about inclusion and services may be stories which display particularly good or bad, or unusual circumstances. These stories have their own importance and validity but if taken on their own may lead to bias on how we respond to inclusion and service challenges. Therefore, accounts of what some term the ‘supercrip’ variety (Kama 2004) may emphasise the strengths of disabled people and may minimise the need for accommodations and services: accounts which emphasise exclusion and dependency (Roulstone 2000) may fail to take adequate account of how services may be changing in a positive direction.

This question of the knowledge basis on which to advocate for better inclusion and improved services relates also to the question of expertise.

## Expertise

It is a fact, and one which is difficult to accept because it is so unjust, that historically disabled people have been excluded from opportunities to develop expertise in research, and in a range of other areas. There are prejudices about what kind of work and thinking disabled people can do - prejudices which persist in Africa (Wolffe, Ajuwon & Kelly 2013): it is not by chance, for example, that the job of a switchboard operator is one which is still associated with blindness in some people’s minds. This exclusion from development, education, training and work must be resisted and questioned, and it is incumbent on all disability researchers to avoid demeaning prejudices and to look for skills and expertise which may be hidden.

In my own work of training disability activists in basic research skills, I was forced to confront my own prejudices, of which I was unaware. One of the exercises we did as part of research training was community mapping, and I assumed that there would be particular challenges with this exercise for blind trainees. It was indeed essential to adapt the exercise to take account of visual impairment, but as a person who does not have a visual impairment, I learned that the trainees who were blind had an excellent understanding of the topography and geography of their home cities. They pointed out that if, as a blind person, one wants to survive life in an African city, one has to know and remember, for example, where vehicles drive on pavements, and where there are potholes and other dangerous obstacles in the road (Swartz 2009; 2010). In fact, blind people may, for reasons of survival, have a better recollection of the topography of their environments. This was a skill and expertise I had not been aware of, but which became obvious through a process which allowed people whom I had underestimated to inform me and others of strengths we did not know they had.

As part of the same training experience, I came to know one trainee who had a 10th grade education. This trainee had been excluded from education partly because of poverty and partly because of disability, and therefore had a low level of functional literacy. This person had been chosen by a national Disabled People’s Organisation (DPO) to be part of the training and was part of a group in which some fellow trainees had master’s degrees. If I had had the opportunity to select trainees for the course, I would probably not have selected this trainee because of his poor educational background. As things turned out, this highly intelligent trainee proved one of the most diligent in the group. He had an excellent understanding of research principles, and I am confident that this understanding was more sophisticated than some fellow trainees with postgraduate qualifications. Through this I learned that I would have been wrong to equate expertise with formal qualifications. I had understood that disabled people are commonly excluded from education but until I had this experience, I had not fully thought through the implications of this in our context: in particular who may have the most to offer in terms of research-mindedness. I have now no doubt in my mind that in future I would choose a research partnership with this ‘uneducated’ trainee long before I would wish to collaborate with some highly qualified researchers.

Having said this, the expertise backlog experienced by this trainee is substantial. The trainee’s level of literacy is such that I very much doubt that without substantial and intensive help he will be able to write a research report unaided in the foreseeable future. I am not an expert on critical periods in development for the establishment of literacy and other skills (Kang, Sarro & Sanes 2014; Lederberg, Schick & Spencer 2013), but I am aware that an enormous amount of work would be needed for this trainee to have a chance of becoming an independent writer in the research field. I wish this was not true, given his obvious intelligence, commitment, and research-mindedness, but the expertise (as opposed to intelligence and potential) is simply not there: exclusion from education had serious consequences in terms of expertise. To deny this is to collude in a rose-coloured vision in which exclusion and oppression are seen not to matter. They do matter, and they do have consequences, even if these are consequences we wish were not there.

At the other end of the spectrum, and as is appropriate, the expertise of formally well qualified researchers to understand disability issues has also been questioned. Winston Churchill famously said of scientists that they should be ‘on tap’ and not ‘on top’: they should use their expertise for the good of society, but they should not be the ones setting the agenda in terms of the research which is needed (Butler 2000). This ‘on tap/on top’ distinction has been used in a number of publications on disability research, and was used as part of the disability knowledge and resources process of scoping disability issues in southern Africa (Albert & Harrison 2005). It is an important ideal that disabled people should themselves be setting the agenda: and the best scenario is that disabled people themselves have all the expertise both to set the research agenda and to do the research themselves. With increasing access to education and training, this is not unrealistic, but currently the situation remains that those on whose behalf much research is carried out and those doing the research are different people. Many apparently simple questions about disability – including, for example, simple ‘how many’ questions about the number of disabled people needing a particular service – involve a reasonably high level of research expertise to address in a meaningful way. Questions need to be asked in particular, answerable ways, and in this regard there is expertise not only in how to conduct and interpret research, but in how to work with researchers to develop such questions which also address priority needs of disabled people.

It is important to recognise that not everybody has the same expertise. Activists are often not trained in research, and many researchers are not good activists. It is a mistake to equate excellent skills in one area with skills in another. As Shakespeare (2013) noted, a confusion between research expertise and activist skills had negative effects on disability research in the United Kingdom: this confusion can have even greater negative effects in a context within which there are huge educational backlogs. An atmosphere of mutual respect in designing and working on research is essential, and it is not a sign of respect to pretend that everybody has the same skills.

In summary, in the dialogues between researchers and disabled people there is still work to be performed in terms of communicating what constitutes research expertise: and this is an issue for the counting of disabled people in research studies.

## Enumeration

I have been at many meetings where participants berated researchers for not having a single definition of disability: I have observed this as a failure by researchers. It has, however, for a long time been known that different ways of measurement may be used to answer very different sorts of questions (Jette 1994). If, for example, we are interested to know about hate crimes perpetrated against people who are viewed as disabled (Sherry 2012), then we need to know about, and to count, people who are perceived by others as disabled. This may be a different figure from people who would self-identify as disabled, and would differ from a count of people who experience certain activity limitations. A key distinction here is between disability as an identity on the one hand, and people experiencing difficulties in doing various activities on the other. It would be likely that people who call themselves disabled would be a much smaller group than those who would say that they have difficulties in certain areas of life, such as mobility, self-care, and social participation.

The Washington Group on Disability Statistics (WG) has carried out and continues to do important work showing that for census and much large-scale research purposes a much broader definition of disability is needed than census formats asked in the past: for example to ask people, ‘Are you disabled?’ (Madans, Loeb, & Altman 2011). The WG advocates measures that are valid and provide accurate estimates of difficulties people have in doing a range of activities. These measures are for use in censuses and population based surveys. The questions developed by the WG enquire about functional or activity limitations rather than about ‘disability’ or being ‘disabled’. Measures of activity limitations facilitate counting all people who have difficulties, such as walking, seeing, hearing, remembering, self-care and communicating, and who may or may not self-identify as being disabled. Disability as an identity would require a separate measure. Asking questions like, ‘Do you have a disability?’ or, ‘Are you disabled?’ do not clearly count either functional limitations or identity and are therefore relatively useless in yielding data that is difficult to interpret (Schneider 2009; Schneider *et al*. 2009).

This useful progress does, however, not come without its own challenges. For example, a recent study on disability and poverty in South Africa used the WG method to ascertain disability status (Graham *et al*. 2014). Included in the sample were elderly people who may be experiencing impairments as a consequence of ageing: therefore some of what was found in terms of the WG method may be a reflection of impairment because of ageing. The authors present data to show that disabled people are more likely than non-disabled people to use public health services, although this may also be a function of age. No other data is presented in this document regarding access to health care, but the authors conclude: ‘The data demonstrated a need for continued efforts to ensure that health services are more accessible for people with disabilities’ Graham *et al*. (2014:2). The authors may have collected data in this regard, but the data is not presented in the document, so it is difficult for any reader to see the basis on which the authors come to this conclusion. The authors show a higher level of health complaints in people they define as disabled as opposed to those they define as not disabled, but they do not show data which deals with access to services: in fact, they show that disabled people have accessed services at a higher rate than non-disabled people. In the presentation of these findings and conclusions we can see the apparent conflation of two things, namely health status, and access to health care. In collecting data on disability, it is important to be exact on what is being measured, and not to conflate one construct with another.

## Evidence

The above discussion about counting raises broader questions about what constitutes evidence. AfriNead is the label for the African Network for Evidence to Action on Disability, but what is this evidence, and who judges its quality? Within mainstream epidemiology – the study of health in populations, and the study of attempts to improve population health – there is a clear hierarchy of evidence, from the anecdote of the single case report at the bottom of the hierarchy, to the evidence generated from a carefully controlled experimental process known as the randomised controlled trial. In this tradition, the best way to know whether something works to change people’s lives is to be able to demonstrate that it indeed is the case, and is generally better than other methods. The field of epidemiology is vast and complex, and a discussion thereof is beyond the scope of this article, but it is important to mention that rigorous epidemiological methods have been applied surprisingly rarely in the context of disability-related research in sub-Saharan Africa. This situation needs attention, and more expertise in epidemiological methods is needed.

This said, it is important to note and understand that there is a politics of evidence. We need to be able to think about what is defined as adequate evidence and who defines the parameters of what constitutes evidence. The definition of the terrain of evidence is in itself an act of power, and issues of power and exclusion of who is claimed to have knowledge and who is not, are at the heart of debates about disability research worldwide. We need to engage with questions on what to make of and how to use, where appropriate, evidence which is seen as characteristically African and not formulated within the dominant international research paradigms (Owusu-Ansah & Mji 2013). The debates about evidence and the politics of evidence are by no means settled (Denzin 2009; Ford & Maher 2013), but the key issue is that the evidentiary base for every claim made in disability research in sub-Saharan Africa (as elsewhere) needs to be explored, before findings and conclusions are accepted.

A key principle of research is, furthermore, that research is evaluated not on the basis of who has performed the study or on whether the findings are similar to those found in other studies, but on the basis of the appropriateness of the methods used, and on whether conclusions drawn are indeed based on the evidence collected. We may agree with the conclusions many researchers draw from their work, but the quality of their work rests on whether those conclusions may be appropriately drawn from the methods and data they have used. In this regard, researchers who change their minds when faced with new evidence which undermines their earlier conclusions, can be regarded as good rather than bad researchers. In very hierarchical social and academic contexts, as occur in our region, it is especially important to be vigilant regarding this issue. In some traditions – often in the practice more than in the theory – things are accepted as more likely to be true and correct because an elder or a leader says so. In the context of research, the quality of evidence depends not on who is providing the evidence but on the quality of the research work. An undergraduate student may well provide a better way of addressing a research problem than a revered senior professor. In this aspect, empirical research traditions have a degree of common cause with disability activists: good researchers are concerned not with traditional status, but with the best possible evidence. Disability activists, at best, are also sceptical of traditional status and are concerned with what makes most sense to improve the lives of disabled people.

## Expectations

It is possible to expect both too much and too little from research and researchers.

On the one hand, it is possible to argue that knowledge is dangerous or useless, as can be seen from the activities of Boko Haram in Nigeria. Even the president of the Republic of South Africa has repeatedly criticised what he terms ‘clever blacks’, implying that they are not authentic Africans (*City Press* 2012). There are good reasons why some disability activists may have low expectations of research and what it can do: as noted at the beginning of this article, there are traditions of research in disability which have led to the exclusion and even abuse of disabled people (Shakespeare 2013). It is probably true, though, that long-standing scepticism about disability research and what it can do has in recent years dissipated, with a more accepting attitude towards it.

This more accepting attitude, however, is not without its own challenges. In introducing its research programme SAFOD, for example, set the following intention: ‘SAFOD aims to become a powerhouse of information and research on disability issues in the SADC region’ (see SAFOD n.d.). This was a noble intention but, in retrospect, it would be a mistake not to recognise that it was overambitious. The hope and expectation that there will be a very dramatic change in the rate and quality of research outputs in the disability research field must be tempered with a realistic assessment of our situation. Resources and expertise are not as freely available as many of us, including myself, would like.

Even where good research is carried out, the expectation that research will change the world also needs to be tempered with realism. Much power of governments rests not on evidence-based policies and practices but on the extent to which policies and practices reflect the wishes and aspirations of various groups, ranging from voters to lobby groups to politicians to donors and to business interests. Good research, especially in a context of compromised governance – which is a reality on much of our continent – will not on its own change the world. It needs to be accompanied by sophisticated and strategic activism. Researchers can provide good data and findings to activists but it is up to activists to use their mobilisation skills to make the research make a difference.

## Concluding comments

These are exciting times for disability research in our region. In exploring the five challenges of experience, expertise, enumeration, evidence and expectations I have suggested that we have much to celebrate but that there is also a long way ahead. At its heart, a research driven approach to disability takes nothing at face value, and it keeps questioning. The more people are involved in thinking about disability research – and the more questions asked – the better for the field and for the realisation of the rights of disabled people.
